# The Transcriptional Network Structure of a Myeloid Cell: A Computational Approach

**DOI:** 10.1155/2017/4858173

**Published:** 2017-09-30

**Authors:** Jesús Espinal-Enríquez, Daniel González-Terán, Enrique Hernández-Lemus

**Affiliations:** ^1^Computational Genomics Division, National Institute of Genomic Medicine, 14610 México City, Mexico; ^2^Centro de Ciencias de la Complejidad, Universidad Nacional Autónoma de México, 04510 México City, Mexico

## Abstract

Understanding the general principles underlying genetic regulation in eukaryotes is an incomplete and challenging endeavor. The lack of experimental information regarding the regulation of the whole set of transcription factors and their targets in different cell types is one of the main reasons to this incompleteness. So far, there is a small set of curated known interactions between transcription factors and their downstream genes. Here, we built a transcription factor network for human monocytic THP-1 myeloid cells based on the experimentally curated *FANTOM4* database where nodes are genes and the experimental interactions correspond to links. We present the topological parameters which define the network as well as some global structural features and introduce a relative inuence parameter to quantify the relevance of a transcription factor in the context of induction of a phenotype. Genes like ZHX2, ADNP, or SMAD6 seem to be highly regulated to avoid an avalanche transcription event. We compare these results with those of *RegulonDB*, a highly curated transcriptional network for the prokaryotic organism *E. coli*, finding similarities between general hallmarks on both transcriptional programs. We believe that an approach, such as the one shown here, could help to understand the one regulation of transcription in eukaryotic cells.

## 1. Introduction

Gene regulation is a key player in the development of living systems. Interactions amongst genes are critical to direct tissue-specific gene expression [1]. A dysfunctional process results in altered physiology, giving rise to malformations and diseases such as cancer. The gene regulatory networks that control gene expression are usually composed by several thousands of genes which are transcribed and translated to produce proteins that have a function in the cell. On the other hand, a reduced set of genes, called *transcription factors* (TFs), has the role of regulating the transcriptional program of the cells. These TFs enhance or repress the expression of other genes, which may be also TFs, or else *target genes*.

By looking at the number of genes that a particular TF could regulate, a hierarchical structure can be observed. In this sense, genes like MEF2C, mTOR, MYB, FOXM1, GATA3, FOXP3, BCL6, MNDA, POU2AF1, MEF2C, or SMAD3 have been reported to potentially regulate more than one thousand genes, which are transcriptional master regulators (TMRs) [2, 3]. The hierarchical character, observed in TF-driven regulatory networks, is likely due to the number of target genes that a TF may have. Another issue related to the relevance of such TFs is whether a given TF is regulated or not by another TF, in which case, a shadow effect could appear, indicating the primacy of a given TF above others [4, 5]. Deregulation of these TFs has been related to the development of cancer and other diseases [2].

**Figure 1:**
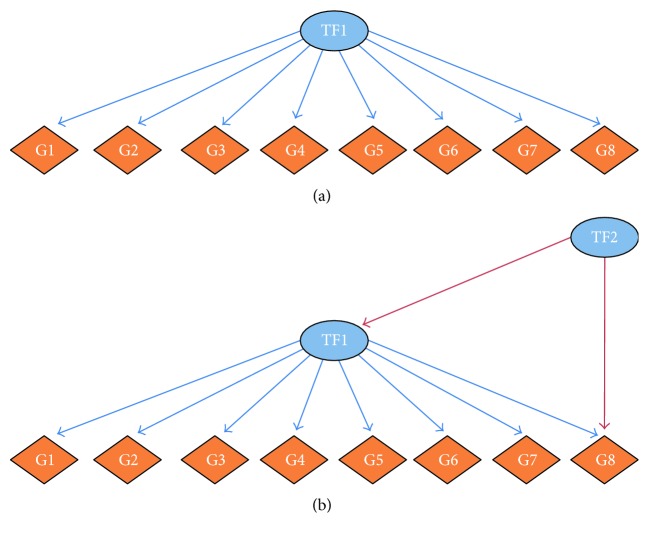
Representative schemes of TFs with high out-degree. (a) The TF1 is regulating its 8 target genes and it is not regulated by others. (b) The TF1 is regulating 8 targets, but at the same time, it is being regulated by TF2, which in turn is also regulating other target genes (G8).

Comprehensive studies regarding the extent of influence of TFs and the targets of the TFs have been made [6]. In such studies, phenomena like feedback, feed-forward loops, and other biological motifs [7] have been found. The said phenomena are indicative of a sophisticated machinery involved in the regulation of gene transcription: highly connected TFs are also regulated by others, which may (or may not) be highly connected. Hence, the importance of TFs in the whole regulatory program lies not only in the out-degree (the number of genes regulated by the TF) but also in the in-degree (how many genes regulate the TF) (Figure 1). It is precisely the interplay of in- and out-degree distributions which shapes ultimately the delicate mechanisms of gene expression control, encoded in the topological structure of the transcriptional regulatory network.

The discovery of general patterns of cooperativity and coregulation for the transcriptional regulation program of eukaryotic cells will help to understand the control of genome-wide expression and how it influences the establishment of phenotypes. To this end, here, we develop a systematic strategy for the data mining curation of the whole set of interactions in *FANTOM4 Edge Express*: a comprehensive and authoritative catalog of transcriptional regulatory interactions in THP1 myeloid cells [1, 8].

We constructed a gene regulatory network for the whole set of genes and their interactions by developing a mining tool for the FANTOM4 database, adding each gene present in the said database as a node to a network, where the strength of connections between nodes corresponds to the intensity, or confidence, of the experimental interaction between any pair of genes. We obtained a network consisting in 9090 genes and 234,913 links. This transcriptional regulatory network contains a number of genes influencing many downstream genes, that is, a few genes are able to control the majority of the genes. As previously mentioned, those TFs are also regulated by other TFs. Taking this into account, we constructed a TF subnetwork, which contains 295 TF genes and 8483 links.

Acknowledging the relevance that in-degree and out-degree of TFs acquire in the context of gene regulation, we devise a *relative influence* parameter (see the corresponding subsection in Materials & Methods), related with the ratio of out-degree/in-degree of all TFs present in the network, thus indicating the relative influence of the gene (and its regulators) in terms of how many targets the said gene is regulating and the number of genes which directly regulate it.

We finally present a network visualization highlighting the importance of those genes. Genes like NRF1, SPIB, GABPA, or TFAP2A present a high RI since they do not have regulators within the database. This could be indicative of a basal and transcription-independent level of regulation, being these genes a kind of default transcriptional regulators. On the other hand, genes like ZHX2, ADNP, or SMAD6 present a very high in-degree, while their out-degree is relatively low. Considering this, we argue that these genes are also relevant, acting as locks avoiding a *transcriptional avalanche*, that is, they can allow the transcription of genes related to rapid cell division or give place to differentiation of the cell. The lack of regulation of them could lead to diseases such as cancer or malformation during development.

As mentioned before, the network also contains experimentally obtained weighted values (*S*_*i*,*j*_) of the interactions between any pair of genes which indicates whether the effect of a gene over any other connected to it is positive (activator) or negative (repressor), that is, there are transcription factors whose “preferential” activity is either of an activating or repressing nature. This fact may follow from the very physicochemical structure of the related protein, its interactions with other proteins and with certain regions in the genome that include, but are not restricted to, the promoter regions on their target genes. Of course, these functions are phenotype dependent and may be even context dependent.

In terms of transcriptional regulation, a positive interaction represents that the source gene (TF) promotes the transcription of the target gene (effector gene). Analogously, a negative interaction will represent that the TF inhibits the transcription of the target gene. The algebraic sum of all interactions of each gene allows us to observe that some TFs are mostly activators and others are mostly repressors.

In the first case, we have for instance NFYA, NFYB, and NFYC; whereas in the latter case, we have TFAP2A, TFAP2C, and MAZ. Arguably, regulation of those genes is fundamental to initiate or terminate a transcriptional cascade, likely related to events of differentiation or cell division. Experiments along this way are necessary. We argue that this framework may help to understand the importance of TFs in terms of the ratio between in- and out-degree, which could give new insights regarding the gene regulation.

## 2. Materials and Methods

Reproducibility of results and methodological clarity are fundamental in all scientific endeavors, but in the case of computational biology approaches, they gain even more relevance. In the present section, we will present detailed accounts of the methods used here, and in some cases (most notably, when we introduce novel concepts), we will even write down detailed calculations. Further details and custom-made computer code for this project are available at the following link: https://github.com/CSB-IG/fantomine.

### 2.1. Network Construction

We mined the FANTOM4 database [8] to construct a gene regulatory network. This database is based on genome-wide dynamics of transcription start site usage in the PMA-stimulated human monocytic cell line THP-1. To this end, we made a systematic approach to search all genes present in the database. We also tracked the genes which regulate the first one as well as the genes regulated by the searched gene. If a gene has no regulators, its in-degree is zero. Analogously, if the searched gene does not have target genes, its out-degree would be zero.

An important source of information about this network is the strength of the interaction between any pair of genes. This interaction is quantified by both experiments and sequence-based transcription factor binding site (TFBS) predictions. The range of intensities *S_i,j_* is a *Z*-score whose dynamic range goes from −10 to 12. With this experimentally obtained interaction value, we constructed a weighted and directed network of the FANTOM4-based gene regulatory network.

The network was built by using a specially devised crawler, following a breadth-first search strategy to find the connections between all genes (promoters and targets). The algorithm begins with a random gene and began to *walk* in the FANTOM EDGE-DB; for each promoter and target of that gene, it adds them to a queue of genes to explore and to a dictionary of the genes explored. If the current gene is new in the dictionary, it adds it to the database and the interaction (if it is a promoter or target); otherwise, it only verifies if the interaction is new or updates the weight (it only takes the biggest value). Since some of the predicted TFBS have different values for the same target depending on the promoter region, we decided to use the highest value for all interactions (for further details regarding the interaction values, please see [8] and the FANTOM4 website: http://fantom.gsc.riken.jp/4/).

As an example of the last sentence, the SRF gene is regulated by SOX2, but this regulation have three different values in the FANTOM database, depending on the promoter region: 4.857, 0.834, and 0.845. We decided to establish a link with the highest value assuming that that interaction is the most plausible in an ideal context.

**Figure 2:**
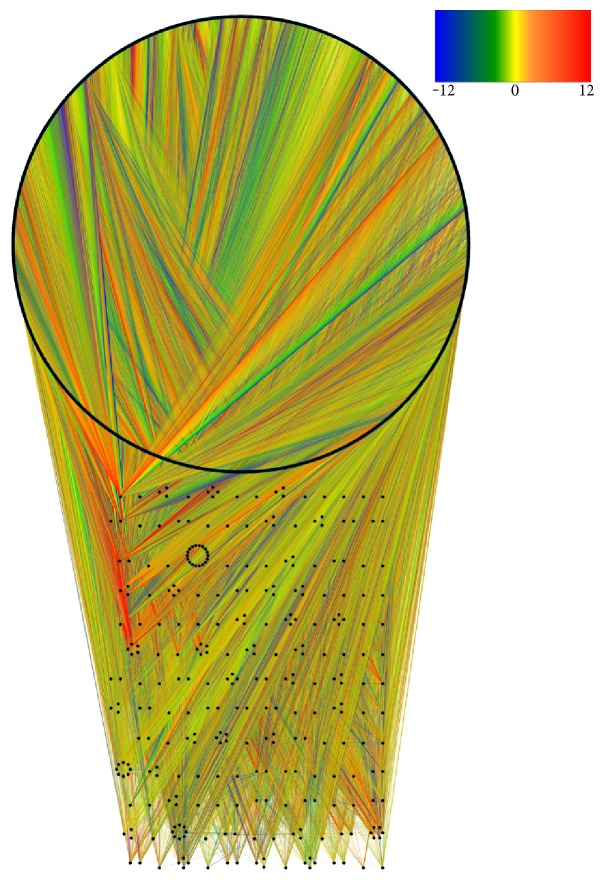
Transcription factor network constructed from the *FANTOM4 Edge Express* database. In this representation, nodes are represented by black dots. The color of the links depends on the value of the interaction: blue and green are repressions; yellow, orange, and red are for activations. The circle on top of the figure corresponds to target genes, that is, their out-degree = 0.

We also calculated different node centrality measures of the resulting network, such as their in-degree and out-degree distributions. The whole network depicted in Figure 2 is visualized by using Cytoscape v.3.2.2.

### 2.2. Relative Inuence

To have a useful measure to retrieve information regarding the regulatory balance of each gene in the context of transcriptional process, we establish a parameter of *relative influence* RI*_n_* for each gene *n* in the network. This parameter reflects the fact that there are some gene regulators that control the transcription of many targets but are in turn regulated by many genes, while other regulators may possess a smaller number of targets but are also regulated by fewer genes and thus may be of similar relative influence on the general transcriptional regulatory program. The RI*_n_* is then obtained as follows:

(1) $$\begin{array}{c} {RI}_{n}=\frac{{outDeg}_{n}}{{inDeg}_{n}+{outDeg}_{n}}-\frac{\in +{inDeg}_{n}}{\in +{outDeg}_{n}}, \end{array}$$

where outDeg*_n_* and inDeg*_n_* are the out-degree and in-degree of the gene *n*, respectively. ∈ is a small variable (10^−3^) to avoid division by zero. We define all negative RI*_n_* values to be set equal to 0.

For the sake of clarity and result reproducibility, we will outline an explicit calculation as follows: For instance, the gene GABPB2 has 232 regulators (in-degree, inDeg) but 2829 targets (out-degree, outDeg) in the database. Hence, RI_GABPB2_ is calculated as follows:

(2) $$\begin{array}{c} {RI}_{GABPB2}=\frac{2829}{232+2829}-\frac{0.001+232}{0.001+2829}=0.842196754. \end{array}$$

Since most genes have zero out-degree, that is, they have no target genes, we constructed a subnetwork which contains only TFs; those are, in general terms, the genes which define the way in which the network is regulated.

### 2.3. The Algebraic Sum of Interactions

Arguably, the interaction strength (*S_i,j_*) of the links in a transcriptional network is an abstract and important parameter to quantify the activity of a TFBS recognition motif on a given TF against all its potential targets, providing information about TF interactions at a gene-by-gene basis. This fact may mask the importance of certain TFs in the transcriptional regulatory programs of the cell.

In order to categorize the importance of different TFs in this network, this value (*S_i,j_*) can be used. A TF may be a transcriptional activator or repressor or a combination of both (for different targets and/or under different circumstances). To clarify this idea, we classify the TFs in terms of the total value of their interaction strength, that is, we sum all *S_i,j_* for each TF*_i_* and observe if the value was positive or negative, under the assumption of an additive linear model. A positive value means the majority of their interactions are positive; thus, TF*_i_* is an *overall activator*. On the other hand, a negative value indicates that the majority of their interactions are for repression; TF*_i_* is acting then mostly as an *overall repressor*. As we have already stated, such terms are context and phenotype dependent, so that a TF whose main function is that of an activator in the context of myeloid cells, it well may be a repressor on a different cell type or cellular context.

Again, as an example, we will outline the explicit calculation for one instance of algebraic sum: TFAP2B has 687 negatively regulated targets and 965 positively regulated ones. The algebraic sum of all their targets is as follows:

∑_*j*_*S*_TFAP2B,*j*_ = 965 − 687 = 278, which converts TFAP2B in a strong *overall activator*.

## 3. Results

### 3.1. Transcription Factor Network

The TF network is shown in Figure 2. 9090 genes with 234,970 interactions are depicted. From that network, we can observe several facts: The network is depicted ranking genes according to the *relative influence*. Upper part genes have a higher RI, meanwhile the lowest RIs are on the lower part. The black circle at the upper part of the network represents those genes with zero out-degree, that is, effector genes. Those genes have RI = 0 by definition. The interactions between genes are weighted according to their respective interaction strength and colored accordingly with a continuous scale, based on the *Z*-scores (*S_i,j_*) for activity expression correlation: this way, the blue and green lines indicate inhibition from the source gene to the target, whereas yellow and red lines represent activation from the source gene to the target (see color scale inset in Figure 2).

We can see, for instance, how a somewhat small set of molecules is responsible for the concerted regulation of the whole transcriptional activity of the cells. The also important fact is that these molecules carry on their regulatory function by jointly regulating themselves. In this network, genes with the highest out-degree are SP1, MAZ, ELF4, ELF1, ELF2, SPI1, ELK1, ELK4, GABPA, and GABPB2. The subnetwork containing only the targets of those genes has 140,318 interactions with 7913 out of the total of genes.

Regarding the RI, the top10 out-degree genes (which are in fact TFs) have high values; however, they do not have the highest ones, since they are also regulated by other TFs.  Table 1 shows those nodes with the highest RI, as well as their experimentally observed expression values at the starting of the experiments. The expression values for the whole time series are provided as a Supplementary material available online at https://doi.org/10.1155/2017/4858173.

As revealed by the RI parameter, TFs with the lowest values have a high out-degree, but at the same time, they present a high in-degree, such is the case of ZHX2, ADNP, SMAD6, POU3F1, GTF2A1, ZIC2, POU6F1, TFAP4, ARID5B, or RUNX1. In all cases, they have (at least) twice more targets than regulators.  Table 2 shows the lowest RI genes.

The *relative influence* is a simple metric which provides relevant information about the regulatory activity of a transcription factor. Interestingly, even when a single instance of RI is not enough to give insight on coregulation and gene expression patterns, the full, genome-wide distribution of RI values does it so. A closer look at the top RI molecules as presented in Tables 1 and 2 unveiled interesting patterns. For instance, amongst the TF genes that are not under regulation (at this level of description, of course, see Table 1), we can find thousands of gene targets.

If we recall that the total number of genes in this study is about 9000, then having TFs regulating around 2000 of these is indeed a powerful indicator of strong coregulation. On the other hand, it is also noticeable that the TF with the lowest RI (ZHX2) although being tightly regulated (with a total of 151 regulators) is able to participate in the regulation of 292 genes, evidencing the cooperative effect in TF regulation.

### 3.2. Transcription Factor Subnetwork

After eliminating all the effector genes (those genes with zero out-degree), the network is drastically reduced (295 genes and 8483 links between them). From this network, it can be observed that some genes are mainly activators meanwhile other TFs are inhibitors. This global activating/inhibiting nature of TFs will be discussed below. Regarding the structure of the subnetwork, it is interesting that several genes are highly regulated, even though they are transcription factors.

We can also see that by considering only the network formed by coregulated transcription factors, it is possible to unveil the presence of modules conformed by groups of TFs that not only regulate other target genes (absent in this network visualization) but also regulate each other. This phenomenon of regulatory modularization is an example of a phenomenon that cannot be seen from the FANTOM4 database consulted on a gene-by-gene basis but provides new biological insight on the whole genome regulatory patterns in this cell lineage.

### 3.3. Some TFs Are Overall Repressors Meanwhile Others Are Activators

**Figure 3:**
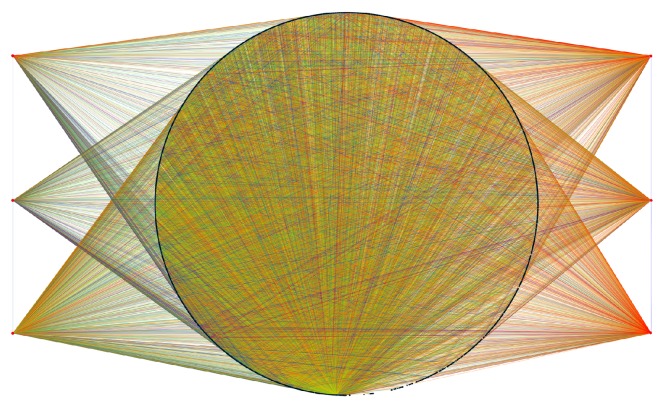
Top 3 overall activating TFs and top 3 overall repressing TFs. On the left side, the three genes which have more inhibiting links—TFAP2C, TFAP2A, and MAZ—are depicted. At the right side, the top 3 activating genes are shown: NFYA, NFYB, and NFYC. It can be observed that the predominance of the green and blue links at the left, meanwhile on the right side, the orange and red links, are more frequent. In the center of this network, we can see the first neighbors of those 6 genes. Links are colored as in Figures 2 and 5.

Taking into account the overall activating or inhibiting nature of TFs (in the context of the cell type and phenotype under consideration), we search in the network those genes with extreme positive and negative values of the sum of interaction strengths (*S_i,j_*) for each TF: They are the most important activators as well as the most important repressors of the transcriptional program. In Figure 3, we show the top 3 activators and also the top 3 repressors along with their targets. Those genes are NFYB, NFYC, and NFYA for activators and MAZ, TFAP2C, and TFAP2A for repressors. The interplay between them may be of importance in shaping the phenotype. Regarding the MAZ gene, it has also a high RI (0.984): This means that it regulates thousands of genes but it is regulated by just a few. It mainly represses the genes that it regulates.

### 3.4. The Transcriptional Network Structure of a Prokaryotic Cell: A Comparison

The topological and functional features, revealed in the transcriptional network of the myeloid cell with the approach performed here, were compared with a prokaryotic model, in order to provide a deeper understanding of the transcriptional regulatory program at the genome-wide level. The prokaryotic network architecture was obtained with the same methodology that we used for the eukaryotic model, but we obtained information about the genetic interactions from RegulonDB [9], a rigorously curated database of a genome-wide transcriptional regulation in the bacteria *Escherichia coli*. This database contains information regarding the type of interaction between genes, positive, negative, or dual, as well as the direction of the said interactions. We decided to analyze the whole transcriptional network, observe the number of transcription factors, calculate the RI for each gene, and obtain the overall activators and repressors.

**Figure 4:**
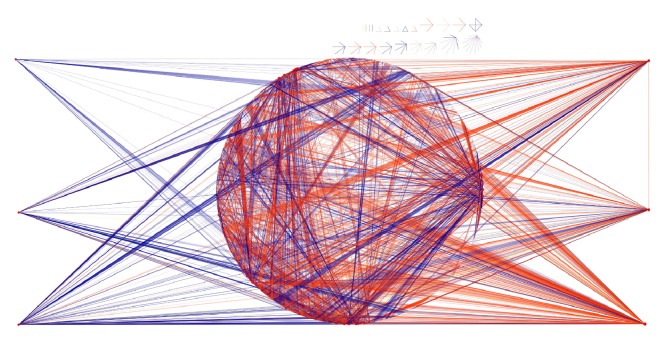
Top 3 overall activating TFs and top 3 overall repressing TFs in the *E. coli* network. On the left side, the three genes which have more inhibiting links—Crp, Fnr, and Fis—are depicted. At the right side, the top 3 activating genes are shown: Narl, Phob, and Lrp. It can be observed (analogous to the human transcriptional network of Figure 5) that the predominance of the blue links at the left, meanwhile on the right side the red links, are more frequent. In the center of this network, the first neighbors of those 6 genes are shown. It is also possible to observe some small disconnected components at the upper part of the figure.

**Figure 5:**
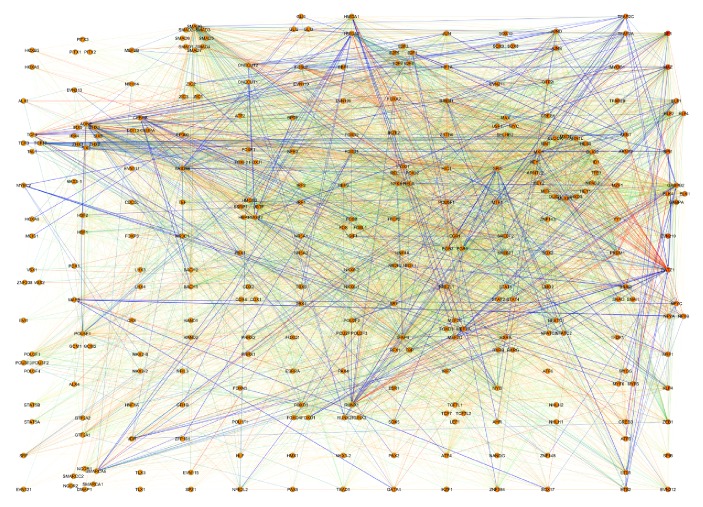
Transcription factor subnetwork. In this graph, the 298 TFs of the FANTOM4 network are depicted. Genes are sorted according to the gene out-degree (top left-to-bottom right); set of nodes describing a circle has the same out-degree. The color code of links is the same as in Figure 2.

This directed network contains 1988 genes and 4414 interactions. 202 of those genes correspond to transcription factors. In Figure 4, we show the transcriptional network of *E. coli*, depicting the links according to its type: blue for inhibition and red for activation. The top 3 overall activators and repressors are separated to illustrate that the general effect that these transcription factors exert on their targets is analogous to Figure 5. Crp, Fnr, and Fis genes are the top 3 repressors, meanwhile Narl, Phob, and Lrp correspond to the top 3 overall activators.  Table 3 shows the top 5 and bottom 5 RI genes of the transcriptional network.

**Figure 6:**
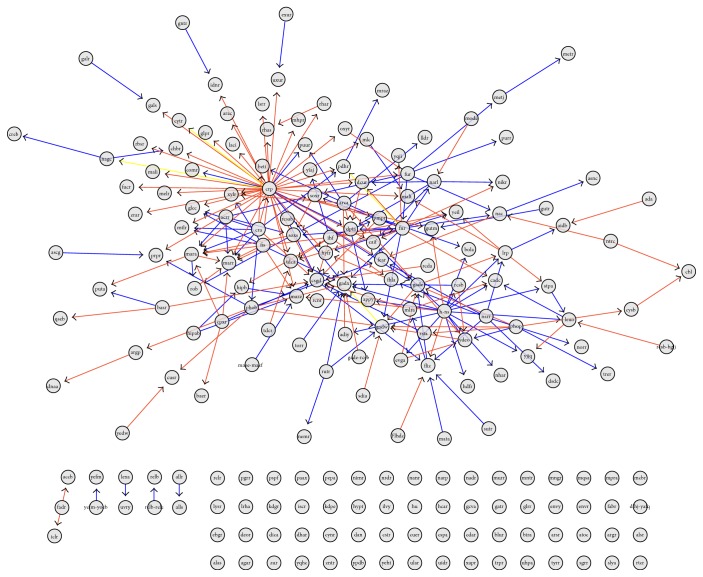
Transcription factor subnetwork. In this graph, the 203 TFs of the *E. coli* RegulonDB network are depicted. The color code of links is the same as that in Figure 4. As it was observed in the whole network, in this TF subnetwork, some disconnected components appear.

We also constructed the TF-only network for *E. coli*. This network is depicted in Figure 6. As it can be observed, coregulation of those TFs is evident.

## 4. Discussion

In this work, we developed a systematic search of the FANTOM4 database, which took into account several experimentally proven interactions between genes and transcription factors. We constructed a network where the genes are nodes and their interactions correspond to links. The network is directed because it takes into account transcription factor binding sites to establish a relationship in which the TF is regulating to another gene (which could actually be another transcription factor). We found several nodes which do not regulate other genes, but they are highly regulated. On the other hand, there is a small group of TFs which are regulating several other genes, but they are not regulated by any gene. This could allow us to hypothesize that those genes are master regulators because they could be inducers of particular phenotypes, as is the case for NRF1, SPIB, or TFAP2A.

### 4.1. The Highest RI Genes May Have Crucial Roles in Cell Specificity

Nuclear respiratory factor 1 (NRF1) [10, 11] activates the expression of crucial metabolic genes related to responses to oxidative stress, endoplasmic reticulum stress, xenobiotic stress, and inflammation [12–14]. NRF1 has also been found misregulated in different carcinomas [15–18]. Despite NRF1 being a key player in the induction of several functions in the cell, there are no reports of transcriptional regulators of NRF1. It is regulated at the translational [19] and posttranslational [19–21] levels. This information reinforces our findings regarding the transcriptional independence of NRF1 as well as the relevance that it acquires in the context of the regulatory network.

The second highest RI belongs to the SPiB gene. The spleen focus-forming virus integration site [22] gene is part of the ETS transcription factor family. It is involved in differentiation [23], immune response [22, 24], apoptosis [25], and activation of early viral expression [26], amongst others. Finally, the third highest RI gene is TFAP2A. This transcription factor is known as a tumor suppressor gene. Decreased expression of this gene has been related to many neoplasms [27–29], as well as other diseases, such as the brachio-oculo-facial syndrome [30, 31].

By observing the relevance of these three genes in the maintenance of correct cell behavior, it is interesting to observe that there are no regulators in our network for those genes. It could be due to the fact that the experimentally curated network is not complete or the available information regarding the regulators is still under construction. However, it is remarkable that the high number of targets contrasting with the number of regulators of them is in instances such as this, where hierarchy acquires relevance. It is worth mentioning that this is a transcriptional network. We are not looking at posttranslational modifications, where these genes could be regulated. However, the transcriptional relevance of them is evidenced by this approach.

### 4.2. Misregulation of the Lowest RI Genes Is Associated with Several Malformations

On the other hand, the three lowest RI genes, ZHX2, ADNP, and SMAD6 are highly regulated; regulation of them is probably more critical in order to avoid transcriptional avalanches. Let us recall that these genes, even though they are highly regulated (higher in-degree counts), are still active transcription factors (see Table 2). In the same sense, mutations of these genes could be involved in the development of several and important diseases. For instance, ZHX2 is considered as a transcriptional repressor [32], which binds the promoter regions, thus regulating transcription of their target genes. This TF also suppresses glypican 3 (GPC3) transcription [33]. Furthermore, ZHX2 has been found as a tumor suppressor [34]. This gene is transcriptionally suppressed by MSX1 and XBP1 [35]. This downregulation is crucial for progression of Hodgkin lymphoma. Concomitantly, there are at least 40 transcription factor binding sites downstream ZHX2 gene [35] which regulate its expression.

The second lowest RI belongs to the ADNP gene, which is essential for brain formation and correct neural development [36]. Mutations in this gene have been related to neuronal disorders such as autism [37, 38], Alzheimer's disease [39], or schizophrenia [40]. Moreover, ADNP interacts with HP1 regulating chromatin remodeling during embryogenesis [41]. Total absence of ADNP is lethal, thus indicating its crucial role in the regulation of transcriptional programs.

Finally, SMAD6 is a signal transducer that modulates multiple signaling pathways, such as the BMP and TGF-beta/activin signaling [42], erythropoiesis [43], or cell cycle [44]. Incorrect regulation of this gene is related to lung adenocarcinoma [42, 45], oral squamous cell carcinoma [46], ovarian cancer [47], or cardiovascular malformation [48].

The RI, introduced here to study the network, remarks the relevance of TFs with the highest values in terms of its master role as transcription factors (since they have no upstream transcriptional regulators); furthermore, this value unveils the importance of the lowest scored genes: those genes need to be highly regulated in order to maintain its correct transcriptional behavior. As it has been observed experimentally, misregulation of the lowest RI genes may result in lethal phenotypes or cancer.

The approach developed here may help to understand genomic regulation. The RI parameter introduced provides insight regarding the influence and importance of the genes in the context of maintenance of a particular phenotype.

### 4.3. Overall Activators and Repressors May Define Transcriptional Programs

Finally, the activation or inhibitory nature of TFs is an important field of investigation. With an approach such as the one presented here, research can be guided to unveil the overall nature of certain TFs, by observing whether its overall effect is activation or inhibition. A TF, which positively regulates hundreds of downstream targets, could induce a particular phenotype by activating the said targets. On the contrary, if the majority of the targets is inhibited by the TF, this gene could prevent or stop a particular transcriptional program.

The following TFs are the top 3 overall activators. Interestingly, this set corresponds precisely with the so-called NF-Y complex: NFYA, NFYB, and NFYC, whose role is to bind a sequence in DNA to start the transcription process. They are involved in several basal activities, such as expression of human proteasome genes [49], transcriptional cascade via CDCA8 gene [50], and remodeling of chromatin [51]. Furthermore, the NFY complex has been associated with the coexpression of other TFs to start transcriptional cascades [52]. However, the separated subunit NFYB also can be an inhibitor of DNA topoisomerase II-*α* [53], indicating that the role of this complex is not restricted to be an activator, revealing thus another aspect of the complexity of the transcriptional program in eukaryotic cells.

As mentioned above, the lowest RI gene in the network is ZHX2. This TF interacts with the A subunit of nuclear factor-Y (NFYA). For instance, ZHX2 represses activation of MDR1 transcription mediated by NFYA [54]. Interestingly enough is the fact that during liver carcinoma, the normal transcriptional program of ZHX2 is highly altered. ZHX2 represses NFYA during liver carcinoma [54]. Taking into account the fact that NFYA is the most important overall activator in the network, we can argue that under a repression of NFYA via ZHX2, the consequence will be a general inhibition of the transcriptional program, which may result for instance, in the progression of liver carcinoma. This example highlights how crucial is the correct control of interconnections in this transcriptional network.

The following are the top 3 overall inhibitor genes and might be involved in the control of transcription by avoiding anomalous events: MAZ, TFAP2A, and TFAP2B. The Myc-associated zinc-finger protein (MAZ) was identified as participating in breast cancer cells by interacting with SAF-1 and inducing transcription of Ras [55]. MAZ also has a role in prostate cancer [56] by interacting with the androgen receptor. On the other hand, TFAP2A, B genes, known tumor suppressor genes, participate in the reduction of glioma progression, by downregulation of Bcl-xl, Bcl-2, c-IAP2, and survivin [27]. These genes have been encountered decreased in several types of cancer, glioma [57], prostate cancer [56], breast cancer [58], or testicular cancer [59]. This is a clear example that the absence of its transcriptional inhibition generates dramatic changes in phenotype.

### 4.4. Similarities with the Prokaryotic Cell Transcriptional Program

Regarding the RI in the *E. coli* network, the higher one, Ihf (integration host factor), plays a crucial role in the survival of the cell, induction of acid resistance, and expression of several other factors [60–62]. H-ns acts on DNA binding of RNA polymerase [63]. The NSrR gene is a major transcriptional repressor in response to iron and also a negative regulator of motility [64–66]. On the other hand, the lowest RI gene is gutM, a crucial transcription factor involved in the phosphotransferase system [67]. Mutations on those genes have a direct impact on the resulting phenotype.

To highlight the importance of the implications that the concepts of RI and the overall activator/repressor may have in the regulatory program, we provide a functional comparison of these measures in the prokaryotic transcriptional network; for this purpose, we can observe (for instance) the Fis gene in *E. coli*. This gene is the second most important overall repressor. Fis acts repressing the Crp gene, which is the most connected gene in the *E. coli* genome. Fis in turn is regulated positively by Ihf, the gene with the highest RI of *E. coli*. With this example, we highlight that the transcriptional regulation of the most influential genes could determine the phenotype of a cell via the transcriptional cascades generated by activation or repression of those influential genes.

### 4.5. Final Considerations

With this approach, we present a hierarchical transcription network built from a highly curated database, containing the values of the interactions between TFs and their targets. We observed that the large majority of genes is controlled by just a few TFs. Those few TFs are strongly coregulated between them, which is translated into a fine tuning in the transcription process. A way to quantify this is by the relative influence parameter (RI), in which the lesser regulated genes and highly regulated ones are relevant for global transcriptional control.

Finally, the extent to which genes are regulated is also important, whether regulation is positive or negative. The negative interaction means that the TF is a repressor of the target, meanwhile a positive interaction represents an activation given by the TF. A global value of each TF in terms of their regulatory values is presented. We observe that NFY subunits are overall activators, meanwhile MAZ and TFAP2A and B are overall repressors. Those genes must be important in the context of transcription. Arguably, regulation of those genes presented in this discussion is fundamental to initiate or terminate a transcriptional cascade, likely related to events of differentiation or cell division. Experiments along this way are necessary. We argue that this framework may help to understand the importance of TFs in terms of the ratio between in- and out-degree as well as their overall effect on targets, which could in turn give new insights regarding the gene regulation.

## Supplementary Material

Supplementary material 1. Expression time series of the Top10 (upper) and Bottom10 (lower) FANTOM4 genes.

## Figures and Tables

**Table 1:** The top 10 highest RI values of TFs in the FANTOM network. The last column shows the average gene expression as given by the number of transcript counts (copy number) at *t* = 0, for each gene.

Gene	In-degree	Out-degree	RI	Avg. transcript count
NRF1	0	2175	0.99999954	1628.215
SPIB	0	1778	0.999999438	316.215
TFAP2A	0	1721	0.999999419	293.865
MYOD1	0	1713	0.999999416	0.02
TFAP2B	0	1652	0.999999395	0.03
ARNT2	0	1627	0.999999385	0.01
SNAI2	0	1543	0.999999352	7.485
MYOG	0	1435	0.999999303	203.985
MYF5	0	1435	0.999999303	0.985
MYF6	0	1435	0.999999303	0.265

**Table 2:** The bottom 10 lowest RI values of TFs in the FANTOM network. The last column shows the average gene expression as given by the number of transcript counts (copy number) at *t* = 0, for each gene.

Gene	In-degree	Out-degree	RI	Avg. transcript count
RUNX1	155	556	0.517749925	9382.91
ARID5B	114	392	0.49206167	12.885
TFAP4	190	632	0.483369442	642.65
POU6F1	87	268	0.442040072	229.68
ZIC2	135	407	0.438502347	10132.46
GTF2A1	100	253	0.357562642	1219.94
POU3F1	80	198	0.331108025	4.585
SMAD6	175	408	0.289214257	2936.75
ADNP	132	292	0.256880007	651.685

**Table 3:** The top 5 highest and lowest values of TFs in the RegulonDB *E. coli* network.

Gene	In-degree	Out-degree	RI
Ihf	0	249	0.999999598
H-ns	0	186	0.999999462
NsrR	0	83	0.999998795
Flhdc	0	80	0.99999875
Narp	0	65	0.999998462

Uvry	1	2	0.166641668
Gadx	15	29	0.141847865
Dpia	6	11	0.101600146
Glcc	4	7	0.064928943
Gutm	4	7	0.064928943

## Additional Points

*Network Data Availability*. Cytoscape .cys files for the networks presented here are available upon request.
