# Supplementary material for: The Transcriptional Network Structure of a Myeloid Cell: A Computational Approach
Source: Int J Genomics. 2017 Sep 30;2017:4858173. doi: 10.1155/2017/4858173 (PMC5651161; doi:10.1155/2017/4858173)
Supplement: Supplementary file 1 — Supplementary material 1. Expression time series of the Top10 (upper) and Bottom10 (lower) FANTOM4 genes. [file 4858173.f1.pdf]

Supplementary material 1. Expression time series of the Top10 (upper) and Bottom10 (lower) FANTOM4 genes.
Each row represents one experimental measure at different time points (bold)

**NRF1**	**0**	**1**	**2**	**4**	**6**	**12**	**24**	**48**	**72**	**96**
Q1(R1)	1912.82	1899.58	1222.65	1925.9	2269.97	1920.06	1088.79	1154.01	1198.74	1101.01
Q1(R3)	1343.61	1080.04	1146.53	1387.4	1497.25	1077.25	900.57	764.5	1016.02	1008.98
	1628.215	1489.81	1184.59	1656.65	1883.61	1498.655	994.68	959.255	1107.38	1054.995

**SPIB**	**0**	**1**	**2**	**4**	**6**	**12**	**24**	**48**	**72**	**96**
Q1(R1)	398.46	355.47	289.65	84.19	93.52	180.02	75.53	65.35	92.32	103.07
Q1(R3)	233.97	199.01	156.02	87.03	63.57	111.69	36.51	46.33	65.08	52.53
	316.215	277.24	222.835	85.61	78.545	145.855	56.02	55.84	78.7	77.8

**TFAP2A**	**0**	**1**	**2**	**4**	**6**	**12**	**24**	**48**	**72**	**96**
Q1(R1)	478.37	404.16	309.58	732.84	829.68	1727.37	850.18	674.92	578.31	543.04
Q1(R3)	109.36	158.76	239.33	553.72	542.11	392.62	341.79	194.9	475.17	505.48
	293.865	281.46	274.455	643.28	685.895	1059.995	595.985	434.91	526.74	524.26

**MYOD1**	**0**	**1**	**2**	**4**	**6**	**12**	**24**	**48**	**72**	**96**
Q1(R1)	0.02	1.42	3.01	1.58	0.89	0.04	0.07	0.05	0.07	0.08
Q1(R3)	0.02	0.03	0.03	1.67	0.03	0.04	0.05	0.04	0.04	0.04
	0.02	0.725	1.52	1.625	0.46	0.04	0.06	0.045	0.055	0.06

**TFAP2B**	**0**	**1**	**2**	**4**	**6**	**12**	**24**	**48**	**72**	**96**
Q1(R1)	0.03	1.62	3.55	0.03	0.03	0.05	0.09	0.07	0.09	0.1
Q1(R3)	0.03	0.04	0.04	0.04	2.06	0.06	5.1	0.05	0.06	0.05
	0.03	0.83	1.795	0.035	1.045	0.055	2.595	0.06	0.075	0.075

**ARNT2**	**0**	**1**	**2**	**4**	**6**	**12**	**24**	**48**	**72**	**96**
Q1(R1)	0.01	0.02	1.27	38.06	83.31	114.4	196.01	318.38	517.44	574.08
Q1(R3)	0.01	0.02	0.61	21.74	35.85	34.6	83.04	135.01	230.47	290.9
	0.01	0.02	0.94	29.9	59.58	74.5	139.525	226.695	373.955	432.49

**SNAI2**	**0**	**1**	**2**	**4**	**6**	**12**	**24**	**48**	**72**	**96**
Q1(R1)	13.21	12.76	130.13	148.05	62.72	21.33	19.77	29.25	16.66	13.72
Q1(R3)	1.76	3.95	20.41	10.53	11.41	3.48	6.63	2.6	8.6	19.77
	7.485	8.355	75.27	79.29	37.065	12.405	13.2	15.925	12.63	16.745

**MYOG**	**0**	**1**	**2**	**4**	**6**	**12**	**24**	**48**	**72**	**96**
Q1(R1)	388.24	109.51	30.37	32.52	71.02	141.62	18.78	39.36	52.82	24.86
Q1(R3)	19.73	7.75	3.23	9.11	10.65	16.58	14.63	8.09	15.46	21.64
	203.985	58.63	16.8	20.815	40.835	79.1	16.705	23.725	34.14	23.25

**MYF5**	**0**	**1**	**2**	**4**	**6**	**12**	**24**	**48**	**72**	**96**
Q1(R1)	1.55	2.4	1.12	1.99	0.98	2.4	1.36	0.92	2.13	1.87
Q1(R3)	0.42	2.24	1.12	1.66	3.72	0.65	1.92	0.03	0.03	1.19
	0.985	2.32	1.12	1.825	2.35	1.525	1.64	0.475	1.08	1.53

**MYF6**	**0**	**1**	**2**	**4**	**6**	**12**	**24**	**48**	**72**	**96**
Q1(R1)	0.51	0.02	0.63	0.74	0.02	0.04	0.06	0.05	0.06	0.07
Q1(R3)	0.02	0.16	0.26	0.55	0.38	0.04	0.05	0.04	0.04	0.04
	0.265	0.09	0.445	0.645	0.2	0.04	0.055	0.045	0.05	0.055

Suppplementary Material 1. Continuation

**RUNX1**	**0**	**1**	**2**	**4**	**6**	**12**	**24**	**48**	**72**	**96**
Q1(R1)	14836.19	11320.35	7309.67	10305.06	9027.64	10290.21	5308.28	4504.74	2146.04	1878.69
Q1(R3)	3929.63	5474.39	4459.87	5377.62	6033.79	4160.26	3347.3	1495.41	2142.14	2288.65

**ARID5B**	**0**	**1**	**2**	**4**	**6**	**12**	**24**	**48**	**72**	**96**
Q1(R1)	17.93	21.36	16.68	223.51	455.36	177.86	180.97	316.66	199.29	84.92
Q1(R3)	7.84	7.45	12.84	64.64	164.68	84.82	121.84	60.22	90.06	124.54

**TFAP4**	**0**	**1**	**2**	**4**	**6**	**12**	**24**	**48**	**72**	**96**
Q1(R1)	791	624.58	530.36	125.02	279.63	525.19	627.86	602.34	776.62	930.29
Q1(R3)	494.3	429.25	203.58	79.28	105.23	176.93	242.32	287.54	326.56	359.03

**POU6F1**	**0**	**1**	**2**	**4**	**6**	**12**	**24**	**48**	**72**	**96**
Q1(R1)	331.95	280.25	227.73	277.84	416.83	945.03	625.01	495.29	440.55	429.07
Q1(R3)	127.41	118.24	128	277.5	478.11	675.39	521.27	591.04	530.11	470.08

**ZIC2**	**0**	**1**	**2**	**4**	**6**	**12**	**24**	**48**	**72**	**96**
Q1(R1)	16560.65	16227.59	21690.27	8556.75	8190.26	8567.82	3919.13	4175.03	3300.68	2981.4
Q1(R3)	3704.27	3467.31	6794.92	3526.95	3453.75	2556.35	1873.26	1320.82	2066.16	2338.12

**GTF2A1**	**0**	**1**	**2**	**4**	**6**	**12**	**24**	**48**	**72**	**96**
Q1(R1)	1921.08	1822.49	1664.75	7503.53	11683.4	7498.26	1549.35	1832.8	963.4	579.14
Q1(R3)	518.8	1059.97	1397.13	3403.57	5047.46	4337.06	2152.05	876.46	961.21	1021.73

**POU3F1**	**0**	**1**	**2**	**4**	**6**	**12**	**24**	**48**	**72**	**96**
Q1(R1)	4.54	2.45	16	15.23	34.74	535.1	677.04	679.59	313.22	183.78
Q1(R3)	4.63	1.53	12.95	10.37	20.81	243.68	367.48	324.86	209.04	158.04

**SMAD6**	**0**	**1**	**2**	**4**	**6**	**12**	**24**	**48**	**72**	**96**
Q1(R1)	4199.09	5196.62	4796.03	3153.54	3233.04	2702.44	2665.38	2907.19	2399.19	2525.92
Q1(R3)	1674.41	1696.32	1588.46	1152.8	1337.97	1353.34	1770.54	1388.1	1738.74	1524.24

**ZHX2**	**0**	**1**	**2**	**4**	**6**	**12**	**24**	**48**	**72**	**96**
Q1(R1)	880.34	247.06	364.73	211.46	397.59	708.21	455.73	277.28	297.07	334.63
Q1(R3)	423.03	288.11	154.05	177.29	276.05	510.54	395.14	157.88	236.27	270.4
